# Exploring separation and reattachment in shear-thinning suspensions through pipe-wall ultrasound measurements

**DOI:** 10.1007/s00348-025-04018-9

**Published:** 2025-04-30

**Authors:** Giuseppe Rosi, Moira Barnes, Frieder Kaiser, David Rival

**Affiliations:** 1 Institute of Fluid Mechanics, Technische Universität Braunschweig, Hermann-Blenk-Straße 23, 38108 Brunswick, Germany; 2 Department of Mechanical and Materials Engineering, Queen’s University, 99 University Ave, Kingston, ON K7L 3N6 Canada; 3 RheEnergise Limited, 5200 Rue Angers, Montreal, QC H4E 1C4 Canada

## Abstract

To better understand how turbulent flow structures develop within shear-thinning suspensions (STSs), we investigate the behavior of a shear layer forming within an STS downstream of a sudden expansion with an expansion ratio of 0.5. Specifically, the shear-layer reattachment behavior downstream of an axisymmetric expansion is characterized through ultrasound imaging velocimetry (UIV) and through pressure measurements, and the observed behavior is used to surmise how the shear layer is modified within the STS. Four fluids are investigated, which include pure water, as well as three 1750 ppm xanthan-gum-in-water solutions mixed with non-reactive mineral microspheres at volume fractions of 0%, 15%, and 30%. Wall-pressure measurements were collected through pressure taps located at 0*h* to 25.8*h* downstream of the expansion with subsequent UIV measurements collected from 1*h* to 9*h* downstream of the expansion, where *h* is the step height and equals the difference between the pipe and throat radii. For single-phase cases, pressure-recovery profiles and UIV flow fields indicate a predictably large reattachment length at low Reynolds numbers, which shortens as the Reynolds number increases from $O(10^2)$ to ${O}(10^4)$ and finally stabilizes at roughly 8*h*. In contrast, the STSs exhibit pressure-recovery and pipe-wall velocity profiles indicating a reattachment length that is consistently short (8*h*) and independent of Reynolds number. The results indicate that the suspended phase within the STSs causes the shear layer to diffuse far more rapidly, thereby promoting momentum transfer toward the wall, which results in a consistently short reattachment length.

**Graphic abstract:**

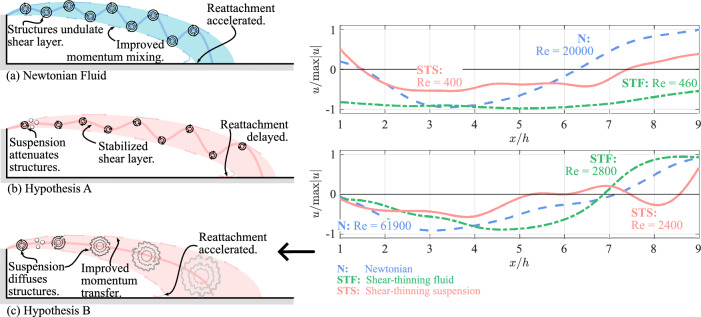

## Introduction

At first glance, turbulent flows involving suspensions in shear-thinning fluids (turbulent STS flows) would appear relevant to a very narrow class of applications. However, such flows have become increasingly relevant to critical areas of research. Beyond typical examples found in the production of petrochemical and foodstuff products (Brennen 2005), turbulent STS flows are central in the development of novel research. For example, the motions of cells or micro-organisms within shear-thinning fluids like mucus or blood can oftentimes be classified as a turbulent STS flow (Pedley and Kessler 1992). Understanding the motion of particles within STS flows is crucial in the development of technologies for sorting target particles or cells in various biological and pharmaceutical processes (Raihan et al. 2020). Turbulent STS flows are also relevant toward understanding the electrochemical and electrorheological properties of certain dense suspensions, which is significant in developing improved energy storage methods (Kupsch et al. 2021).

Given the absence of work done on turbulent STS flows, one may infer their behavior by considering turbulent flows involving somewhat related flow media: a dense suspension of solid particles carried by a Newtonian fluid; and a single-phase shear-thinning fluid. In the case of dense suspensions, statistics of canonical turbulent flows such as pipe and channel flow would suggest a “leveling-out” or smearing of turbulence, with regions that would present with low Reynolds stresses in a single-phase fluid exhibiting an increase in Reynolds stress, and vice versa. This has been observed in turbulent channel flows involving dense suspensions where, relative to a Newtonian fluid, the inner layer exhibits an increase in Reynolds stresses, while the Reynolds-stress peak within the outer layer reduces (Costa et al. 2018; Fornari et al. 2016). In pipe flows, this turbulent smearing within dense suspensions has caused the elimination of turbulent spots that would, in a single-phase flow, instigate turbulence (Hogendoorn and Poelma 2018), while in a von Kármán swirling flow (a.k.a French washing machine) turbulent attenuation has been observed (Cisse et al. 2015). In the case of shear-thinning fluids, simulations of pipe flows (Singh et al. 2017) and experiments in sudden expansions (Castro and Pinho 1995; Escudier and Smith 1999; Pak et al. 1990) have shown a general reduction in Reynolds stresses, whereas experimental results of concentric annuli show no remarkable changes in the Reynolds stresses (Escudier et al. 1995).

The mechanisms causing the aforementioned changes in turbulence statistics within both dense suspensions and shear-thinning fluids may be better understood by characterizing the changes experienced by the underlying turbulent coherent structures (TCSs). This has certainly been the case in Newtonian fluids, where hairpin vortices have been shown to be significant in explaining aspects of wall-bounded turbulence including transition, near-wall statistics and the perseverance of low-speed streaks (Adrian 2007; Wallace 2012). Unfortunately, work on TCSs within these more complex flow media remains limited. Statistical analyses of the motions of suspended particles, as well as their surrounding velocity fields, indicate the tendency of particles to participate in hairpin-vortex-induced ejection events and to accumulate in the wake of hairpin vortices (Berk and Coletti 2020; Kiger and Pan 2002; Richter and Sullivan 2014). These same studies show a weakening of the hairpin vortices by the disperse phase, but their findings are limited by the severely low volume fractions that were tested. Other studies performed at higher loading fractions indicate that the elimination of maxima within the wall-normal profiles of Reynolds stresses and turbulent kinetic energy are caused by the disruption and elimination of typical TCSs, but provide little evidence or mechanistic explanation as to why (Fornari et al. 2016; Patro and Dash 2014; Picano et al. 2015). More progress has been made with regard to the modification of TCSs within shear-thinning and viscoelastic fluids. Direct numerical simulations that have assessed the effect of shear-thinning rheology on TCSs in wall-bounded flows show that TCSs appear elongated, weakened and with a reduced number density (Arosemena et al. 2021; Saeed and Elbing 2023), all which speak to the reduction in turbulence intensity observed in the above-mentioned studies. Similar observations have been made with regard to jets of viscoelastic fluid, with instabilities along the jet’s periphery appearing larger, elongated, weaker and less frequent (Guimarães et al. 2020).

**Fig. 1:**
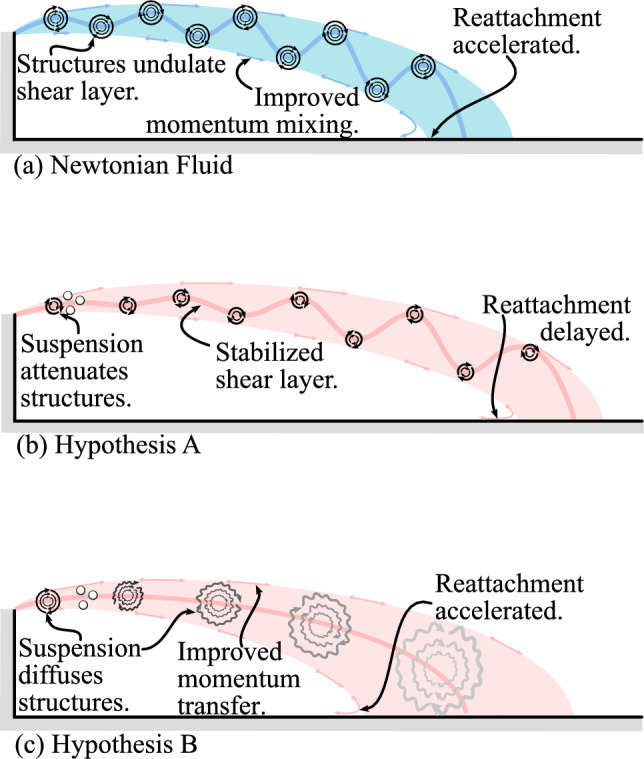
**a** For a Newtonian fluid passing through a sudden expansion, the reattachment point convects further downstream with increasing Reynolds number. This proceeds until $\text{Re}\sim {O}(10^2)$, at which point instabilities form within the shear layer, promoting momentum mixing toward the wall and accelerating reattachment. **b** In the case of a shear-thinning suspension, the attenuation of instabilities by the suspended phase may result in increased shear-layer stabilization, thereby delaying reattachment. **c** Alternatively, the rapid diffusion of vortical structures might improve momentum transfer toward the wall, thereby accelerating reattachment

The intractability of turbulence coupled with the added complexity of an STS likely makes understanding TCS modification within complex flows involving STSs a difficult avenue to pursue. Instead, it may be more fruitful to investigate how STSs modify coherent structures in isolation, apply those findings to quantify TCS modification within more complex flows, and finally describe the mechanisms behind turbulence modification in STS flows. A good candidate to study is the flow downstream of a sudden expansion. The flow comprises of two counter-rotating vortices that are encased by the wall and a shear layer that sheds from the expansion’s lip. The distance between the expansion and the point where the shear layer reattaches with the wall (i.e., the reattachment length) is of particular interest, since it is determined by the shear layer’s stability and topology. In a Newtonian flow, the reattachment length grows with Reynolds number when $\text{Re}\lesssim {O}(10^2)$, since increasing the flow speed convects the shear layer further downstream but the rate at which momentum spreads toward the wall remains constant through viscous diffusion. The reattachment length reaches a maximum at approximately ${O}(10^2) \lesssim \text{Re} \lesssim {O}(10^3)$, at which point Kelvin–Helmholtz instabilities and shear-layer flapping develop within the shear layer, which promotes momentum transfer toward the wall and ultimately accelerates reattachment (see Fig. 1a). The strength of the instabilities and consequentially momentum mixing grow with Reynolds number, thereby reducing the reattachment length when $\text{Re}$ increases from ${O}(10^3)$ to ${O}(10^4)$. Finally, at $\text{Re} \gtrsim {O}(10^4)$, the forward convection of the shear layer and the momentum mixing toward the wall equalize, the flow becomes self-similar, and the reattachment length stays constant (Back and Roschke 1972).

Others have examined sudden-expansion flows involving dense suspensions and shear-thinning fluids. In the case of shear-thinning fluids, studies have shown that the fluid’s anisotropic behavior attenuates turbulent disturbances in the stream-normal (i.e., radial) direction. However, despite this turbulence attenuation, the same studies observed roughly equal reattachment lengths to Newtonian fluids over a broad Reynolds-number range (Castro and Pinho 1995; Escudier and Smith 1999; Pak et al. 1990). The tendency of shear-thinning fluids to attenuate stream-normal disturbances has been attributed to the viscous relaxation of the fluid in the streamwise direction, resulting in the accentuation of streamwise turbulence and the attenuation of turbulence in other directions. It should be mentioned that Pereira and Pinho (2000) observed a shorter reattachment length for shear-thinning fluids relative to Newtonian fluids. They attributed their observation to a greater level of turbulence prior to the expansion in comparison with other experiments.

The behavior of dense suspensions downstream of a sudden expansion remains a far-less explored topic. Time-averaged simulations that have treated the suspension as a continuum have observed reattachment lengths in line with Newtonian fluids for $\text{Re}\sim {O}(10^2)$ (Wu et al. 2020), while others have observed improved pressure recovery with increased volume fraction, which was attributed to increased particle–particle collisions (Senapati and Dash 2020). Recent experiments have revealed that increasing the volume fraction of the suspended phase reduces large-scale turbulent mixing (Jeronimo and Rival 2021), which may coincide with results showing a rapid diffusion and attenuation of the vortical shear-layer structures forming within dense suspensions (Barnes et al. 2024; Zhang and Rival 2020).

It remains difficult to infer the reattachment length for an STS being transmitted through a sudden expansion, even in light of past findings involving shear-thinning fluids and dense suspensions. On the one hand, since the carrier fluid of the STS is shear-thinning, one might predict a reattachment length consistent with Newtonian fluids despite how shear-thinning fluids present with fewer and weaker turbulent coherent structures. Less information is available on TCS modification within Newtonian dense suspensions, and it is unclear how the “leveling-out” of turbulence that is typically observed in dense suspensions might affect the reattachment length. One might argue that the suspended phase might promote a longer reattachment length by reducing the strength of the instabilities within the shear layer, which could have a stabilizing effect (see Fig. 1b). One could also argue that the suspended phase will promote a shorter reattachment length by rapidly diffusing the shear layer toward the wall (see Fig. 1c). The current study seeks to explore this question by experimentally investigating the reattachment length of STSs downstream of a sudden expansion.

Experiments involving STS flows can be quite challenging using standard optical methods. These challenges have given rise to alternative velocimetry measurement techniques that do not require unobstructed optical access. Current state-of-the-art methods include magnetic resonance velocimetry (MRV) (Elkins and Alley 2007) or X-ray velocimetry (Mäkiharju et al. 2022), both of which are unobstructed by the presence of a disperse phase. Ultrasonic imaging velocimetry (UIV) also shares in the same benefits, but avoids challenges such as affordability, complex post-processing and the development of test facilities devoid of ferromagnetic materials as faced by MRV; or regulatory issues and the complexity involved with disentangling the integrated measurement along the beam axis as faced by X-ray velocimetry (Poelma 2016). UIV has been successfully employed toward the investigation of various dense suspensions. Examples include Kupsch et al. (2021), who investigated narrow-channel flows common in operational zinc–air batteries and revealed a flat-top velocity profile and increased wall-slip; Gurung et al. (2016), who revealed flow fields within opaque drilling fluid; Han et al. (2019), who investigated “particle jamming” phenomena in dense suspensions upon solid impact; and Crapper et al. (1998) who demonstrated the suitability of UIV toward studying sediment flows relevant to oceanography and ocean engineering. A thorough review of UIV along with additional examples can be found in Poelma (2016).

As stated, the current study seeks to characterize how TCSs modify within an STS flow by investigating a relatively simple and predictable flow structure, i.e., a shear layer reattaching downstream of a sudden expansion. By quantifying the reattachment behavior, the study seeks to answer if the “leveling-out” behavior or the turbulent-attenuation behavior of the suspended phase dominates the reattachment phenomenon. Toward this end, UIV is applied in concert with pressure measurements to identify the reattachment point downstream of a sudden axisymmetric expansion of an STS fluid, and to investigate the point’s correlation with the fluid’s pressure recovery. The following section describes the experimental setup, including the tested fluids; the test facility and the expansion; the pressure measurements; and the UIV apparatus. Results are then discussed, which include the pressure-recovery measurements for all tested fluids; imaging data gathered by the UIV setup; and flow fields and reattachment lengths as determined through the UIV. Finally, the implications of the results as it pertains to reattachment length and pressure recovery are summarized, and the successes and limitations of UIV in measuring the tested fluids are discussed.

## Methods

The following section first describes the properties of the fluids tested within the sudden expansion. The flow-loop facility and the geometry of the sudden expansion are then discussed. This section then describes the pressure sensors and their locations along the test section, as well as the UIV apparatus and its resultant field of view. Finally, the test matrix of the measurement campaign is laid out in terms of the volume fraction of the disperse phase and in terms of the throat velocity. The throat velocity is presented nominally and in terms of an equivalent Reynolds number described herein.

### Properties of tested fluids

Pressure and UIV measurements were taken in water, as well as in three 1750 parts-per-million (ppm) xanthan-gum-in-water solutions mixed with insoluble non-reactive mineral microspheres ($\rho _p>4000\,\text{kg/m}^{3}$). The three mixtures formed STSs with particle-volume fractions of ${\Phi }=0\%$, $15\%$, and $30\%$, respectively. For brevity, these four fluids will be, respectively, referred to as water, XG${\Phi }0\%$, XG${\Phi }15\%$, and XG${\Phi }30\%$. To assess the viscoelastic behavior of the shear-thinning fluid, a series of relaxation and oscillatory tests were performed using characteristic shear rates and angular frequencies derived from the step height and throat velocities tested herein. The results revealed a relaxation time of approximately 10 s and a viscous-to-elastic modulus of unity, indicating that the fluid exhibits moderate viscoelastic properties. It is also noted that previous studies on the behavior of shear-thinning fluids through sudden expansions have employed similarly concentrated xanthan-gum solutions at comparable Reynolds numbers (Escudier and Smith 1999; Pereira and Pinho 2000, 2002).

**Fig. 2:**
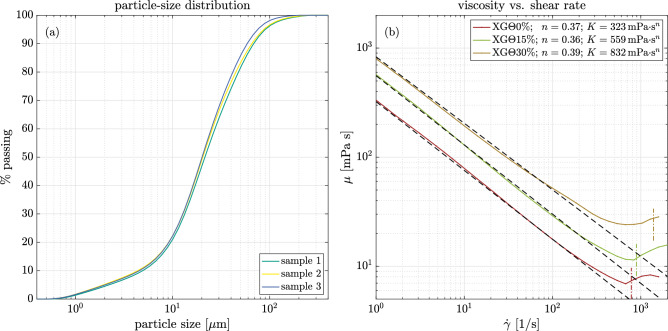
**a** Particle-size distribution of three random samples of the mineral particles in the dense suspension. **b** Viscosity curves of the shear-thinning fluids: XG${\Phi }0\%$, XG${\Phi }15\%$, and XG${\Phi }30\%$, with solid curves, dashed lines, and vertical dash-dotted lines indicating viscosity measurements, power-law fits, and the onset of Taylor–Couette instabilities, respectively

Figure 2a shows the particle-size distribution of three independent samples that were assessed using a Horiba Partica Laser Scattering Particle Size Distribution Analyzer LA-950V2. The mean and median diameters of the particles are 31.5 $\upmu$m and 20.7 $\upmu$m, respectively. The small particle size in combination with the dissolved viscosifier (xanthan gum) lead to small Stokes numbers and avoided settling of the suspensions in between experiments. The fluids were weighed to determine their density ($\rho$), while an Anton Paar MCR302 Rheometer was used to characterize the viscosity ($\mu$) of the fluids. Figure 2b shows the viscosity as a function of the shear rate for XG${\Phi }0\%$, XG${\Phi }15\%$, and XG${\Phi }30\%$, respectively. Until the onset of a Taylor–Couette instability in the rheometer’s cylinder-cup geometry (marked with vertical dashed-dotted lines in Fig. 2b), the data can be described using power-law coefficients (*K* and *n*) in the form $\mu =K\dot{\gamma }^{(n-1)}$ (dashed lines), where $\dot{\gamma }$ is the shear rate. Increasing the volume fraction of solids has little impact on the shear-thinning behavior (slope $n\approx \text{const}$). However, denser suspensions exhibit an increased *K*, leading to an upward shift of the viscosity curves. Densities and effective viscosities were ultimately used to calculate the effective Reynolds number ${\text{Re}}_{\mathrm{eff}}=\rho U_t d/\mu _{\mathrm{eff}}$, where $\dot{\gamma } = 2U_t/(D-d)$ was used to calculate $\mu _{\mathrm{eff}}$. In these previous expressions, $U_t$ is the throat velocity, *D* is the pipe diameter, and *d* is the throat diameter. It is noted here that $(D-d)/2$, which is the characteristic length used to calculate $\dot{\gamma }$, is termed the step height and is denoted by *h*. The methodology used here to estimate the Reynolds number is consistent with previous studies on non-Newtonian flows through sudden expansions (Castro and Pinho 1995; Pereira and Pinho 2000; Poole and Escudier 2004).

The density is also used to calculate the static-pressure coefficient $\sigma ={\Delta } p /(0.5\rho U_t^2)$, which normalizes the difference between downstream pressure and throat pressure (${\Delta } p$) with the kinetic energy content of the flow $(0.5\rho U_t^2)$. An overview of the fluid properties is provided in Table 1.

**Table 1:** Densities and power-law coefficients of tested fluids

Fluid	$\rho$ (kg/${\text{m}}^3$)	*K* (mPa ${\mathrm{s}}^n$)	*n* (unitless)
Water	998	0.89	1.000
XG${\Phi }$0%	998	322.9	0.369
XG${\Phi }$15%	1450	559.0	0.365
XG${\Phi }$30%	1955	832.4	0.390

### Flow-loop facility

**Fig. 3:**
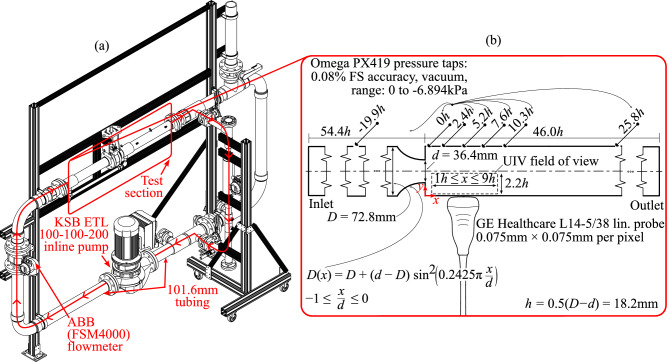
Schematics of: **a** the low-pressure-flow-loop (XLPFL) facility indicating the pump, flowmeter and test section; and **b** the test section indicating relevant geometry, pressure-tap locations, and the UIV field of view

Measurements were taken in RheEnergise’s HTC2.0 facility within the low-pressure flow loop (XLPFL). The flow loop is shown in Fig. 3a. The loop is constructed primarily out of 101.6-mm tubing and circulates fluid using a KSB ETL 100-100-200 inline pump. Flow rates are recorded by an ABB (FSM4000) flowmeter, while density measurements were periodically performed via an inline Promass I-300 Coriolis flowmeter. Pressure and UIV measurements were captured within the test section shown schematically in Fig. 3b. The test section comprised of an axisymmetric expansion with a throat diameter (*d*) of 36.4 mm and a pipe diameter (*D*) of 72.8 mm resulting in a diameter ratio of $\beta =0.5$ and a step height of $h=18.2$ mm. The throat of the expansion is located 54.4*h* downstream from the inlet of the test section, while the outlet of the test section is located 46.0*h* downstream from the expansion’s throat. The contraction occurs gradually over a distance of a single throat diameter, the profile of which is provided as an equation in Fig. 3b. Previous pressure measurements involving various Newtonian and non-Newtonian fluids within laminar and turbulent regimes have confirmed the flow upstream of the throat is fully developed and without secondary flows.

The experimental apparatus used here emulates previous studies (Back and Roschke 1972; Escudier and Smith 1999; Pereira and Pinho 2000, 2002), where an extended pipe develops the flow upstream, leading to a contraction and then a sudden expansion. When the results of these previous studies are compared to those from Pak et al. (1990), who employed a settling chamber followed by an abrupt contraction into an expansion, it is apparent that boundary-layer development has a very limited effect on the results. Instead, fluid type and shear-layer dynamics play a much larger role (Pereira and Pinho 2000, 2002).

Eight-millimeter-diameter taps equipped with pressure sensors (Omega PX419, 0.08% accuracy, vacuum, range: 0 to − 6.894 kPa) were placed at one location upstream of the throat, as well as six locations downstream of the throat as indicated by Fig. 3b from which the static-pressure coefficient $\sigma ={\Delta } p /(0.5\rho U_t^2)$ was calculated. The taps were 16.25 mm deep and were connected to their respective sensor using 1-m-long Tygon tubes. Each tubing system was equipped with a valve to facilitate a siphon that would flush the line with water, preventing air bubbles or test fluid from entering into the tube. The tubes were carefully checked for contaminants prior to pressure logging and would be flushed again when necessary.

UIV measurements were taken using a GE Healthcare L14-5/38 linear probe with 128 elements and a 0.3-mm pixel pitch, coupled with a Vantage 128 ultrasound system from Verasonics. The Vantage system allows for plane wave imaging by simultaneously activating all elements within the ultrasound probe, thereby achieving high temporal resolutions. Tilted plane wave imaging was used to improve image quality and ultimately resulted in a spatial resolution of 0.075 mm by 0.075 mm per pixel. The field of view captured by the ultrasonic probe spanned a streamwise distance of 2*h* in the streamwise direction and penetrated a radial distance of 2.2*h* into the pipe. The UIV probe was traversed along the pipe to capture four separate fields of view that were stitched at $x/h=3$, 5, and 7, resulting in a single mean flow field spanning a streamwise domain of $1 \le x/h \le 9$. To mitigate discontinuities, mean velocities were interpolated across the vertical seams using spline interpolation, followed by five iterations of Savitzky–Golay smoothing applied to the stitched velocities and their first adjacent neighbors. Adjacent fields of view were positioned with an accuracy within the vector spacing (0.065*h*), resulting in a reattachment-length uncertainty of 0.2*h*.

For measurements involving water, $10\,{\upmu m}$-diameter contrast agent bubbles (DEFINITY^®^, Lantheus) were seeded into the flow as tracer particles, while for measurements involving XG${\Phi }0\%$, air bubbles were used as tracer particles. Both tracers exhibited a Stokes number far beneath unity during all experiments, ensuring tracer accuracy errors less than 1%. Lastly, for cases involving XG${\Phi }15\%$ and XG${\Phi }30\%$, the mineral particles functioned as tracer particles. At worst, the Stokes number of the mineral particles roughly equals unity, corresponding to tracer accuracy errors of approximately 1%. The Stokes number is estimated here using the following definition adopted after Joseph et al. (2001):

1 $$\begin{aligned} \text{Stk}=\dfrac{1}{9}\dfrac{\rho _p}{\rho _f}\dfrac{d_p}{h}{\text{Re}}_{\mathrm{eff}}, \end{aligned}$$

### Test matrix

The test matrix of the measurement campaign is summarized in Table 2. The leftmost column presents tested fluids, while the second and third columns present the nominal throat velocity and effective Reynolds number, respectively. Pressure measurements were taken for all listed cases. However, UIV measurements were attempted only for the boldfaced cases. It is noted here that the characteristic shear rates for all test cases fall within the rheometer-data ranges that are well described by the power-law fits.

**Table 2:** Test matrix summary: pressure measurements were taken for all cases, whereas UIV was attempted for boldfaced cases only

Fluid	$U_t$ (m/s)	${\text{Re}}_{\mathrm{eff}} /10^3$
Water	**0**.**51**, 1.01, **1**.**52**, 3.00, 4.52	**20**.**6**, 41.3, **61**.**9**, 122.6, 184.7
XG${\Phi }$0%	**0**.**51**, 1.01, **1**.**52**, 3.00, 4.52	**0**.**46**, 1.43, **2**.**8**, 8.5, 16.6
XG${\Phi }$15%	**0**.**51**, 1.01, **1**.**52**, 3.00, 4.52	**0**.**40**, 1.2, **2**.**4**, 7.2, 14.1
XG${\Phi }$30%	**0**.**51**, 1.01, **1**.**52**, 3.00	**0**.**33**, 1.0, **1**.**9**, 5.7

## Results

The current section first focuses on pressure recovery downstream of the sudden expansion for all tested fluids. The implications that the fluids’ pressure recoveries have on their respective reattachment lengths are then discussed. Finally, the different fluids’ reattachment lengths estimated through UIV are presented and compared to their respective pressure recoveries.

### Pressure recovery and shear-layer implications

**Fig. 4:**
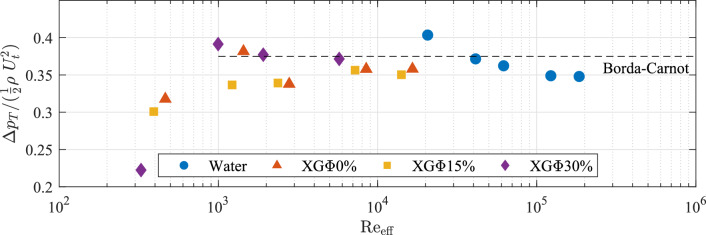
Total pressure-recovery coefficient (${\Delta } p_T /(0.5\rho U_t^2)$) for all tested cases. All cases are plotted against their effective Reynolds number, while the markers indicate different fluid types. The Borda–Carnot prediction is indicated by the dashed line. The total pressure-recovery coefficient demonstrates no discernible trend with effective Reynolds number or fluid type, but is consistently within 10% of the Borda–Carnot prediction

Figure 4 presents the total static-pressure-recovery coefficient ${\Delta } p_T /(0.5\rho U_t^2)$ for all four fluids as a function of the effective Reynolds number $\text{Re}_{\mathrm{eff}}$. The dashed-line represents the Borda–Carnot prediction and is given by the equation:

2 $$\begin{aligned} {\Delta } p_{\mathrm{BC}} =2\beta ^2\left( 1-\beta ^2\right) \left( \frac{1}{2}\rho U_t^2 \right) \end{aligned}$$

Besides two outliers, all cases fall within 10% of the Borda–Carnot prediction and exhibit no meaningful trends with effective Reynolds number or fluid type. Tashiro and Tomita (1984, 1986) also observed strong agreement with the Borda–Carnot prediction across a variety of different flow conditions for air-solid suspensions. The results presented herein would suggest that neither the disperse phase nor the shear-thinning nature of the continuous phase have a significant effect on the total pressure recovery downstream of the expansion for $\text{Re}_{\mathrm{eff}}>{O}(10^3)$.

**Fig. 5:**
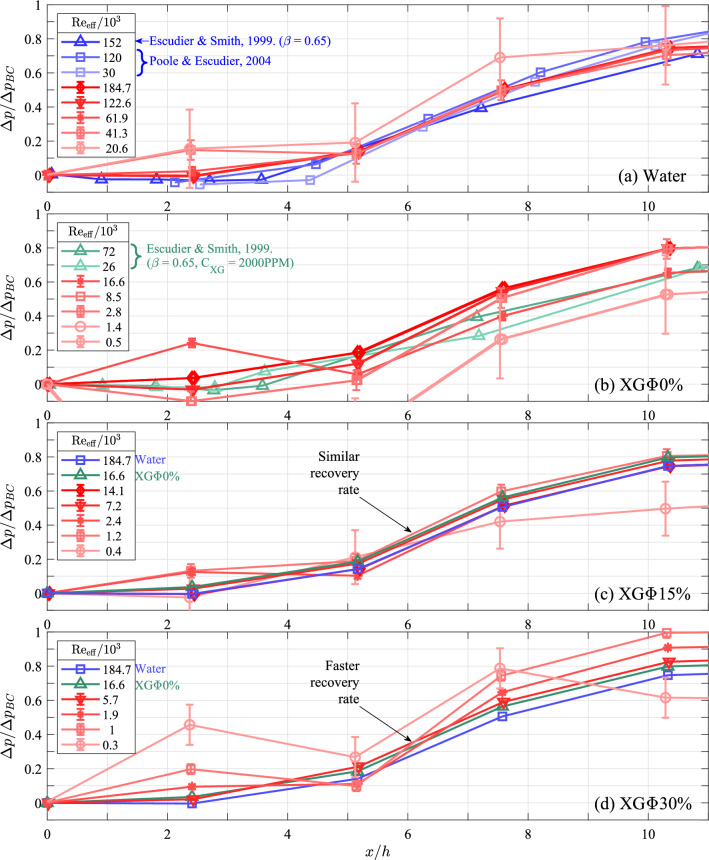
Pressure recovery normalized by the Borda–Carnot prediction $({\Delta } p /{\Delta } p_{\text{BC}})$ plotted against downstream distance from the expansion’s throat normalized by the step height (*x*/*h*), for **a** water, **b** XG${\Phi } 0\%$, **c** XG${\Phi } 15\%$ and **d** XG${\Phi } 30\%$. The legends list the effective Reynolds numbers of cases ($\mathrm{Re}_{\mathrm{eff}}$). For the sake of comparison, relevant single-phase results from Escudier and Smith (1999) and Poole and Escudier (2004) are included in (**a**) and (**b**), while in (**c**) and (**d**), the highest $\mathrm{Re}_{\mathrm{eff}}$ cases for water and XG${\Phi }0\%$ are included

The rate at which the pressure recovers downstream of the expansion provides a measure of the reattachment length, since the length of the recirculation zone controls the rate at which the fluid expands and repressurizes. Figure 5 presents pressure-recovery profiles by plotting normalized static-pressure change $({\Delta } p /{\Delta } p_{\mathrm{BC}})$ as a function of downstream distance (*x*/*h*). Error bars indicate the propagated uncertainty of the pressure measurements. At the lowest Reynolds number, small pressure differences and weak orifice dynamic pressures lead to higher uncertainty, whereas all higher Reynolds numbers exhibit stronger signals and lower uncertainty. Each pressure measurement was obtained by averaging 60,000 sequential samples collected at 1 kHz for 60 s at each tap. The resulting values of ${\Delta } p / {\Delta } p_{\mathrm{BC}}$ have a maximum standard error of 2% based on the standard deviation of the pressure measurements. Measurements stabilized within 1% of their statistical means after 20 s worth of data, indicating convergence. Additionally, repeated trials confirmed that pressure measurements remained within the uncertainty of the sensors, demonstrating repeatability.

Generally, all four fluids exhibit little pressure recovery prior to 2*h* downstream. This is followed by a rapid increase in pressure recovery that culminates to roughly 80% of the Borda–Carnot prediction by 10*h*. For comparison, relevant pressure-recovery measurements from Escudier and Smith (1999) and Poole and Escudier (2004) of single-phase fluids downstream of a sudden expansion have been overlaid onto the single-phase fluid results (Fig. 5a, b). Furthermore, the highest Reynolds-number cases for water and XG${\Phi }0\%$ have been overlaid onto the STS results (Fig. 5c, d).

The pressure recovery of water (Fig. 5a) exhibits no discernible trend with Reynolds number and agrees well with previous results from literature. The result is expected given the high Reynolds numbers $(\text{Re}_{\text{eff}}>{O}(10^4))$ and the fact that the reattachment length stabilizes at $\text{Re}_{\text{eff}}\sim {O}(10^4)$ for Newtonian fluids (Pak et al. 1990). In contrast, for cases involving $\text{XG}{\Phi }0\%$ (Fig. 5b), the rate of pressure recovery appears to increase with Reynolds number, ultimately overtaking the pressure recovery observed in Escudier and Smith (1999). The increase of the pressure-recovery rate with Reynolds number agrees with previous studies and is indicative of the destabilization of the shear layer and shortening of the reattachment length that generally occurs within the tested $\text{Re}_{\text{eff}}$ range (Castro and Pinho 1995; Pak et al. 1990; Pereira and Pinho 2000). Some high-$\text{Re}_{\text{eff}}$ cases exhibit a faster pressure recovery in comparison with that observed by Escudier and Smith (1999). These faster pressure-recovery rates may have been caused by differences in diameter ratio or shear-thinning agent concentration, but the exact cause is uncertain.

The pressure-recovery profiles of the STSs at different Reynolds numbers are plotted in Fig. 5c and d. The highest $\mathrm{Re}_{\mathrm{eff}}$ cases for both water and XG${\Phi }0\%$ are included in both plots for comparison. All XG${\Phi }15\%$ cases present with pressure-recovery profiles that roughly match the overlaid single-phase cases. This is an especially interesting result considering that within a single-phase flow, one would expect some Reynolds-number dependency on the reattachment within the tested $\mathrm{Re}_{\mathrm{eff}}$ range of XG${\Phi }15\%$. Instead, all XG${\Phi }15\%$ cases present with pressure recoveries that would suggest a reattachment length that is independent of $\mathrm{Re}_{\mathrm{eff}}$ and equal to that found for a single-phase flow within the self-similar turbulent regime. The XG${\Phi }30\%$ cases present with even faster pressure-recovery rates that overtake the highest $\mathrm{Re}_{\mathrm{eff}}$ single-phase cases. Here, the XG${\Phi }30\%$ cases reach $\sim 70\%$ of the Borda–Carnot prediction by 7.6*h*, whereas the single-phase cases reach a similar level of pressure recovery by 10.3*h*. The result suggests that the XG${\Phi }30\%$ cases present with reattachment lengths that are even shorter than the single-phase fluids within the self-similar turbulent regime.

We quickly address the lowest $\mathrm{Re}_{\mathrm{eff}}$ cases for XG${\Phi }15\%$ and XG${\Phi }30\%$, as they appear seemingly as outliers. The pressure recovery of these two cases fall beneath that of the single-phase cases further downstream, which may appear to suggest a slower pressure recovery. However, the pressures attained in both these cases by 10.3*h* closely resemble their total pressure recovery measured at 25.8*h*, indicating that both cases have already fully expanded and instead have experienced a pressure loss relative to the Borda–Carnot prediction. It is speculated that the heightened pressure losses exhibited by these two cases are caused by particle-wall collisions, which has been shown to sizably contribute to pressure loss at lower Reynolds numbers (Patro and Dash 2014). Furthermore, it is noted that across most cases, the tap at 2.2*h* tends to indicate positive pressure recovery relative to the throat. This is an observation not typically seen in previous studies, as the region immediately downstream of the expansion usually exhibits a reduction in pressure relative to the throat (Escudier and Smith 1999; Khodaparast et al. 2014). One possible explanation for this anomalous result is the presence of a wall-normal velocity component contributing to the static-pressure measurement, either due to the saddle between the primary and secondary recirculation regions or due to a slight misalignment in the tap itself.

The pressure-recovery profiles within the STS cases can be used to provide commentary with regards to the underlying shear-layer behavior. The pressure recovery for XG${\Phi }15\%$, which is mostly identical across a Reynolds-number range where the reattachment length is Reynolds number dependent for single-phase fluids and matches the pressure recovery of single-phase fluids within the self-similar turbulent regime suggests that the disperse phase has a dominant effect on the shear-layer’s behavior. This is further reinforced by the pressure recovery observed for XG${\Phi }30\%$, which overtakes the pressure recovery of the single-phase fluids despite XG${\Phi }30\%$ being tested within $\mathrm{Re}_{\mathrm{eff}}$ range where single-phase fluids present with large reattachment lengths. The faster pressure recoveries of the STSs would suggest that the presence of the suspended phase promotes an early reattachment of the shear layer. It is doubtful that the suspended phase would have a destabilizing effect on the shear layer thereby promoting reattachment through an early onset of turbulence, especially given how the suspended phase tends to weaken TCSs in canonical turbulent flows and diffuses coherent vortical structures in isolation. Instead, it is speculated that this quick reattachment is caused by the “leveling-out” effect of the suspension, causing the shear layer to rapidly diffuse and reattach more quickly due to momentum transfer toward the wall, as depicted in Fig. 1a.

The results presented in this section show that STSs have little effect on the total pressure recovery downstream of a sudden expansion for $\text{Re}_{\text{eff}}>{O}(10^3)$. Furthermore, the STSs present with faster pressure-recovery rates, which suggest quick reattachment of the shear layer. We attempt to corroborate the second finding through the use of UIV data collected downstream of the expansion. The following section describes the UIV data used to identify the reattachment point and in turn discusses these results.

### Determining reattachment from UIV data

**Fig. 6:**
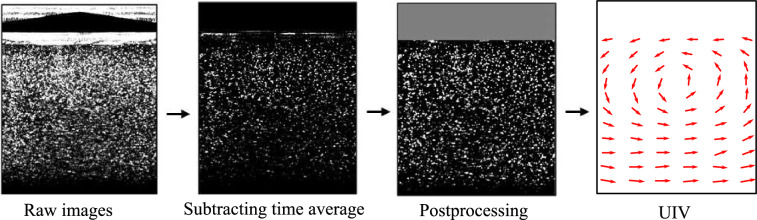
Image-processing procedure performed to prepare ultrasound-rendered images for flow-field extraction

Figure 6 presents the typical image-processing procedure used to prepare ultrasound-rendered images for flow-field extraction. A raw image generated by the ultrasound probe is shown in Fig. 6a. To reduce noise, a time average is subtracted from the raw images, resulting in post-processed images like that shown in Fig. 6b. Additional post-processing measures are then taken, such as locally normalizing the intensity of the image to mitigate non-uniform brightness, as well as masking. These final images are then processed in DaVis 11, generating two-dimensional flow fields such as that presented in Fig. 6d. The reader is referred to Najjari et al. (2021) for further details regarding the image-processing procedure.

**Fig. 7:**
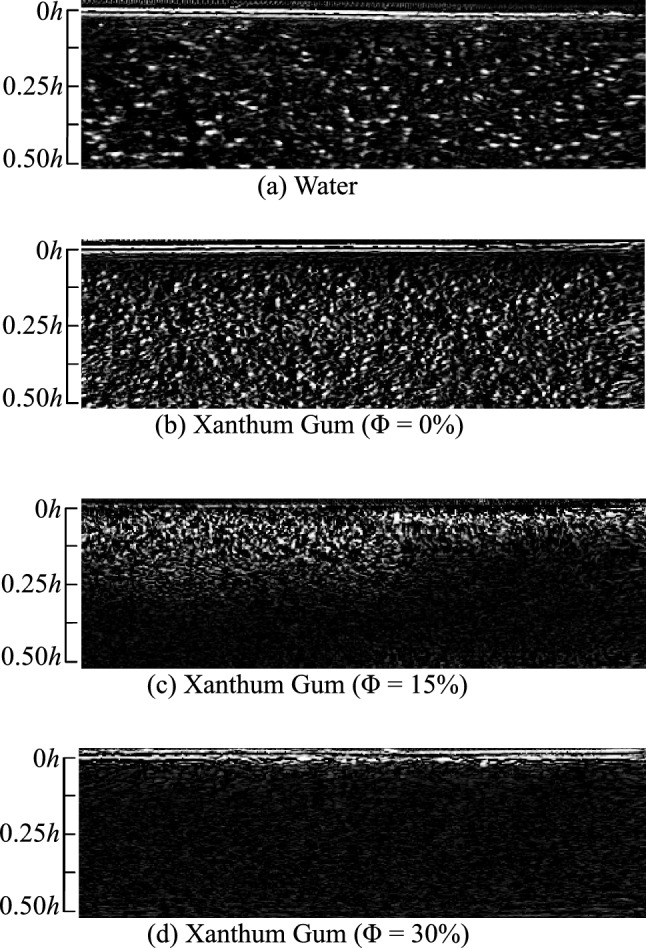
Ultrasound images taken near the pipe wall (top) for **a** water **b** XG${\Phi }0\%$, **c** XG${\Phi }15\%$ and **d** XG${\Phi }30\%$. The pipe wall is located along the top edge and flow is from left to right. A scale is provided in each image indicating depth into the pipe

UIV data for the cases outlined in Table 2 were collected. Typical post-processed images zoomed in near the pipe wall are shown in Fig. 7 for all fluids. Within these images, the pipe wall is located along the top edge and flow runs left to right. A scale is provided in each image indicating depth into the pipe. For both water and XG${\Phi }0\%$, the absence of a disperse phase results in very clear particle images that are amenable to flow-field measurements. However, for XG${\Phi }15\%$ and XG${\Phi }30\%$, the disperse phase’s difference in density and the presence of trapped air causes scattering and attenuation of the sonic signal, which in turn reduces the signal-to-noise ratio. This is clear when comparing the speckle images of water and XG${\Phi }0\%$ to those for XG${\Phi }15\%$ and XG${\Phi }30\%$. In the case of XG${\Phi }15\%$, the ultrasound could reliably image particles a distance of only 0.125*h* from the pipe wall. Despite this reduction in image quality, the images for XG${\Phi }15\%$ provided sufficient coherence to assess the direction of the flow near the pipe wall, which was used to determine the reattachment point. The images for XG${\Phi }30\%$ were ultimately too incoherent to extract any meaningful data, which speaks to the limitation of tilted plane wave UIV when measuring in particle-laden flows with very large volume fractions and large density differences between phases.

**Fig. 8:**
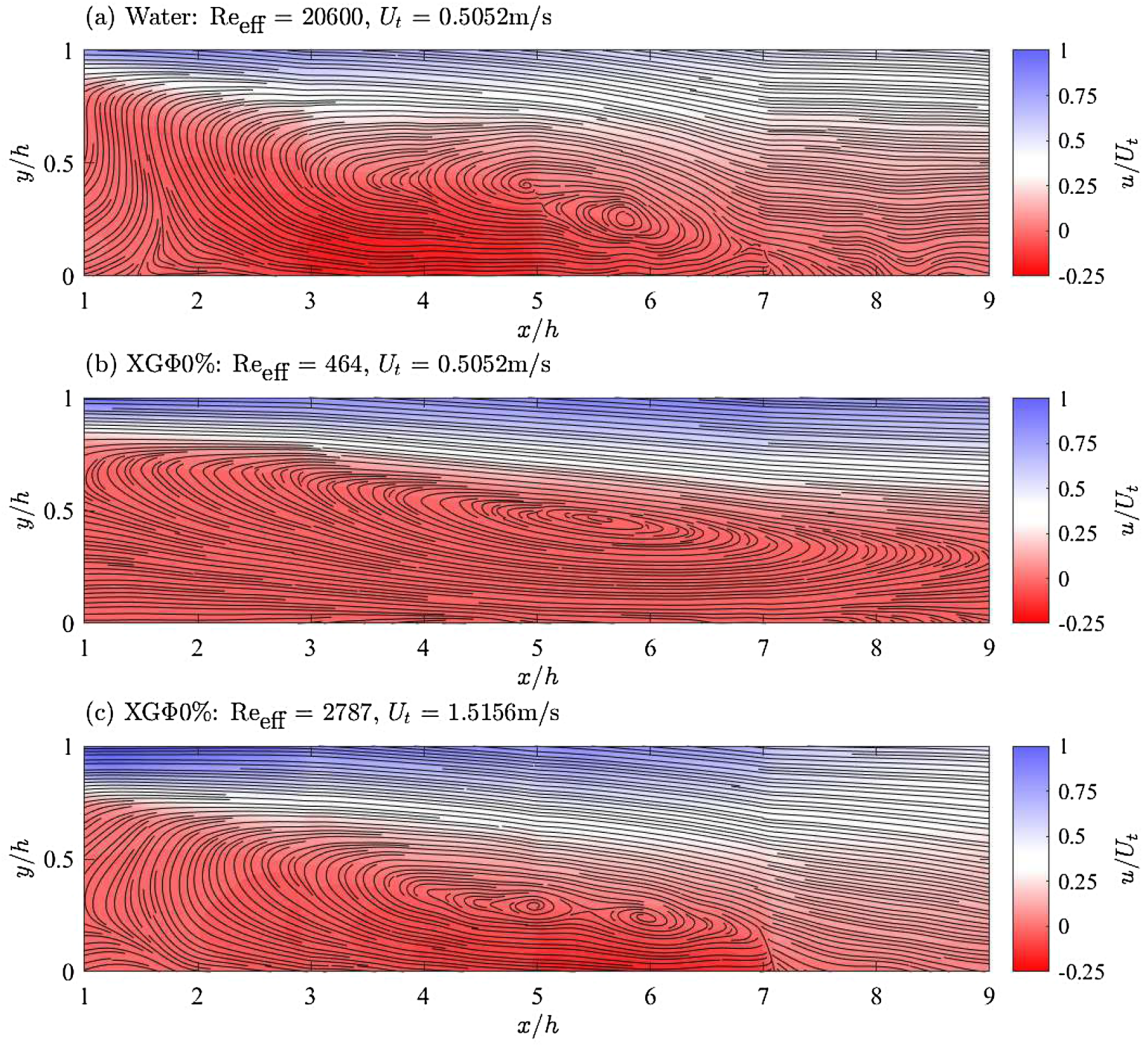
Fields of streamwise velocity normalized by the throat velocity ($u/U_t$) for single-phase water and xantham-gum solution ($\text{XG}{\Phi }0\%$) with streamlines overlaid to highlight the recirculation region and reattachment. The throat velocity and effective Reynolds number of the cases are indicated within the caption titles of each plot. **a** For water at $\text{Re}_{\text{eff}}=20{,}600$, streamlines would indicate a reattachment length of roughly 8*h*. **b** $\text{XG}{\Phi }0\%$ at $\text{Re}_{\text{eff}}=464$ presents with a reattachment length beyond the field of view. **c** When $\text{Re}_{\text{eff}}$ of $\text{XG}{\Phi }0\%$ is increased to 2787, the reattachment point moves upstream to roughly 8*h*

Figure 8 presents mean flow fields collected via UIV for the single-phase (water and XG${\Phi }$0%) cases. UIV data for cases with throat velocities of $U_t=0.5052$ m/s and 1.5156 m/s were, respectively, sampled at 265 Hz and 800 Hz, resulting in particle displacements of approximately 7 pixels/frame, which is ideal for PIV experiments and ensures minimal uncertainty (Raffel et al. 2018). Mean velocity fields and pipe-wall velocities were calculated from 2449 samples of the instantaneous field. The resulting streamwise and vertical velocity components exhibited global standard errors of 2% of the throat velocity, indicating low random uncertainty. The streamwise velocity exhibited a residual of 1% relative to the local cumulative mean after averaging 2000 samples, suggesting convergence. In contrast, after the same number of samples, the vertical velocity component exhibited a residual of 10%, indicating incomplete convergence. This difference is primarily due to the small magnitude of the vertical velocity, typically 100 times smaller than the streamwise velocity, which led to a poor signal-to-noise ratio and reduced accuracy in its measurement. This lack of convergence, as well as the uncertainty in the flow-field seaming procedure may have contributed to artifacts in the streamline field, particularly the dual vortex cores observed in Fig. 8a and c.

Figure 8a presents the mean streamwise-velocity field (*u*) downstream of the throat for water at an effective Reynolds number of $\text{Re}_{\text{eff}}=20{,}600$. Streamlines have been superimposed to reveal the mean flow. The streamwise (*x*) and spanwise (*y*) dimensions are normalized by the step height (*h*), while the streamwise velocity is normalized by the velocity at the throat ($U_t$). The throat is located at $x/h=0$, and the central axis of the pipe is located at $y/h=2$. UIV for water at $\text{Re}_{\text{eff}}=61{,}900$ revealed a nearly identical flow topology and is excluded here. In both cases, the flow topologies indicate a reattachment length of roughly 8*h*. Previous studies have also observed a stable reattachment point in Newtonian fluids at $\text{Re}_{\text{eff}}\gtrsim {O}(10^4)$, with reattachment lengths ranging between 8 and 12*h* (Back and Roschke 1972; Castro and Pinho 1995; Escudier and Smith 1999; Pak et al. 1990; Pereira and Pinho 2000; Poole and Escudier 2004). The stable reattachment point revealed by the UIV for water corresponds well with water’s stable pressure-recovery profiles presented in Fig. 5a. As discussed previously, the consistent pressure-recovery profiles in water were indicative of a constant reattachment length across all tested $\text{Re}_{\text{eff}}$. The UIV measurements presented here, as well as observations made by previous studies, suggest that all tested water cases exist within the self-similar turbulent regime and exhibit reattachment lengths of roughly 8*h*.

Figure 8b and c presents similar flow fields as in Fig. 8a but for XG${\Phi }$0% at effective Reynolds numbers of $\text{Re}_{\text{eff}}=464$ and 2787, respectively. The streamline topology for XG${\Phi }$0% at $\text{Re}_{\text{eff}}=464$ suggests that the reattachment length is located beyond the field of view at around 9*h*. Upon increasing the Reynolds number from $\text{Re}_{\text{eff}}=464$ to 2787 (Fig. 8b), the reattachment length moves to roughly 8*h*. The reduction in reattachment length within the tested Reynolds-number range agrees with previous studies (Castro and Pinho 1995; Escudier and Smith 1999; Pak et al. 1990; Pereira and Pinho 2000) who generally report the reattachment length reaching a maximum at $\text{Re}_{\text{eff}}\sim {O}(10^2)$, followed by the reattachment length decreasing and ultimately stabilizing after $\text{Re}_{\text{eff}}\gtrsim {O}(10^3)$ to ${O}(10^4)$. The reduction in the reattachment length corresponds well with the previously discussed pressure measurements for XG${\Phi }$0% (Fig. 5b), in which XG${\Phi }$0% exhibited a delayed pressure recovery when $\text{Re}_{\text{eff}}=464$ relative to XG${\Phi }$0% at higher Reynolds numbers. As described previously, the delayed pressure recovery for XG${\Phi }$0% at $\text{Re}_{\text{eff}}=464$ is indicative of a delayed reattachment, while the faster and more consistent pressure recoveries for XG${\Phi }$0% at higher $\text{Re}_{\text{eff}}$ indicate a reduction and stabilization of the reattachment length. The flow fields presented here appear to corroborate this and would suggest that XG${\Phi }$0% is approaching a stable reattachment length of roughly 8*h* as the Reynolds number increases. It is mentioned here that the double-vortex patterns seen in the recirculation zones of Fig. 8a and c have not been observed in similar studies (Escudier and Smith 1999) and is likely caused by vortex-core meandering, as well as a poor signal-to-noise ratio of the small vertical velocities within the recirculation zone.

**Fig. 9:**
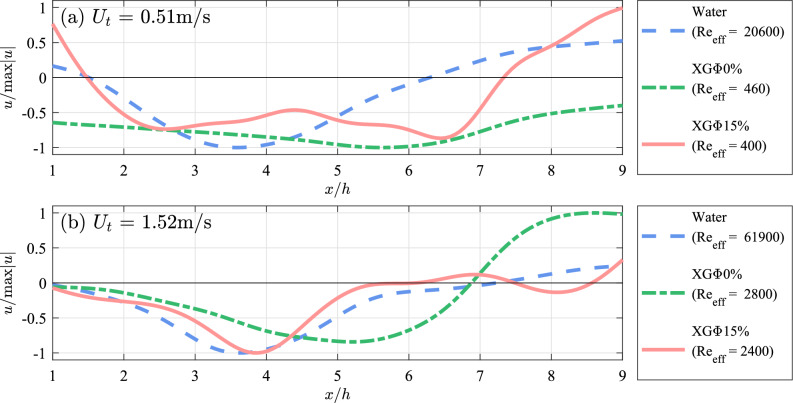
Streamwise velocity along the pipe wall normalized by the maximum streamwise pipe-wall velocity magnitude for **a** $U_t =0.51$ m/s and **b** 1.52 m/s. $\text{Re}_{\text{eff}}$ are provided for each fluid case within the legends. The point where the pipe-wall velocity transitions from negative to positive provides an indication of the reattachment length

In the case of XG${\Phi }15\%$, the reattachment length was assessed by identifying the location where the mean pipe-wall velocity changed sign. The pipe-wall velocity was taken as the first row of time-averaged streamwise velocity vectors within the pipe generated by the UIV measurements. The results of this exercise are presented in Fig. 9. Specifically, Fig. 9a and b respectively show the streamwise pipe-wall velocity of all fluids for throat velocities of $U_t = 0.51$ m/s and 1.52 m/s. Here, velocities have been normalized by the maximum streamwise-velocity magnitude measured along the pipe wall. It is observed that velocity switches from negative to positive at $x/h \approx 7$ for both cases involving water. For XG${\Phi }0$%, the velocity does not switch sign at the lower velocity, but does cross at $x/h \approx 7$ for the higher velocity. The result is consistent with the reattachment lengths identified through streamlines in Fig. 5, which speaks to the reliability of the method in accurately identifying the reattachment point in the absence of flow fields.

With regard to XG${\Phi }15$%, when $\text{Re}_{\text{eff}}$ equals both 400 and 2400, the pipe-wall velocities generally exhibit an increasing trend and, based on the location of sign change, indicate a similar reattachment length between 6*h* and 8*h* despite the magnitude difference in Reynolds number. This consistent and short reattachment length coincides with that for both water and XG${\Phi }0\%$ at high Reynolds numbers, and suggests an absence of Reynolds-number dependence as observed in the single-phase cases. The reattachment lengths for XG${\Phi }15\%$ also agree with the fluid’s corresponding pressure measurements in Fig. 5c. Specifically, all XG${\Phi }15\%$ cases exhibited pressure-recovery profiles that were in line with the highest Reynolds-number single-phase cases. The UIV presented here, as well as the pressure recovery, suggest that STSs present with short reattachment lengths that are independent of Reynolds number.

## Discussion

Relying on the reported pressure and ultrasound measurements, as well as on previous results from literature, we discuss here the reattachment behavior of STSs and the implications this behavior has on the embedded shear layer. In the single-phase cases studied herein (water and XG${\Phi }0\%$), both the pressure and ultrasound measurements indicated a reattachment behavior consistent with previous studies. Specifically, the results indicate a reattachment length in single-phase fluids that elongates when $\mathrm{Re}_{\mathrm{eff}}$ increases toward $\sim {O}(10^2)$, but then reduces and finally stabilizes to roughly 8*h* when $\mathrm{Re}_{\mathrm{eff}}$ reaches $\sim {O}(10^3)$. This general trend in reattachment length observed here within single-phase fluids is consistent with previous studies (Back and Roschke 1972; Castro and Pinho 1995; Escudier and Smith 1999; Pak et al. 1990; Pereira and Pinho 2000; Poole and Escudier 2004) and is directly related to the shear layer’s behavior as the Reynolds number increases. As described in Sect. 1, the shear layer convects further downstream with increasing $\mathrm{Re}_{\mathrm{eff}}$, causing the reattachment to reach a maximum at $\mathrm{Re}_{\mathrm{eff}}\sim {O(10^2)}$. Further increases in $\mathrm{Re}_{\mathrm{eff}}$ causes the shear layer to destabilize and undulate through the formation of Kelvin–Helmholtz instabilities and shear-layer flapping. The destabilization accelerates reattachment through heightened momentum mixing toward the pipe wall, resulting in a reduction in reattachment length when $\mathrm{Re}_{\mathrm{eff}}$ increases from ${O(10^2)}$ to ${O(10^3)}$. The reattachment length stabilizes at roughly $\mathrm{Re}_{\mathrm{eff}}\sim {O(10^3)}$ to ${O(10^4)}$, which is the point where the competing effects of forward shear-layer convection and wallward momentum exchange equalize and result in a steady reattachment.

The STSs tested here (XG${\Phi }15\%$ and XG${\Phi }30\%$) did not exhibit characteristics of a flow with a reattachment process that was Reynolds-number dependent. Instead, both the pressure and ultrasound measurements indicated a reattachment length that was independent of Reynolds number and comparable to what is observed in single-phase shear layers within the self-similar turbulent regime. Specifically, the pressure-recovery profiles of the shear-thinning suspensions were similar across all tested cases, with XG${\Phi }15\%$ and XG${\Phi }30\%$ , respectively, exhibiting pressure-recovery rates that matched and even overtook the highest $\mathrm{Re}_{\mathrm{eff}}$ single-phase cases. Furthermore, the ultrasound measurements showed reattachment lengths in XG${\Phi }15\%$ that were independent of Reynolds number and were roughly equal to that of the single-phase cases in the turbulent regime.

Based on previous studies on single-phase shear-thinning fluids and dense suspensions, Sect. 1 tried to predict how the shear layer would behave within an STS. Previous studies on the reattachment of shear-thinning fluids downstream of a sudden expansion show very little change in the shear layer’s reattachment length relative to a Newtonian fluid (Castro and Pinho 1995; Escudier and Smith 1999; Pak et al. 1990; Pereira and Pinho 2000), despite a consistent attenuation of both the turbulence and the underlying coherent structures in these and other studies (Arosemena et al. 2021; Guimarães et al. 2020; Saeed and Elbing 2023).

Studies on dense suspensions in various flow environments have shown an attenuation of coherent turbulent structures and a “leveling out” of turbulence in wall-bounded turbulent flows, two characteristics which have has been reported in a general sense by Patro and Dash (2014), Picano et al. (2015), Fornari et al. (2016) and Costa et al. (2018); specifically for the attenuation of hairpin vortices by Kiger and Pan (2002) and Berk and Coletti (2020); and for velocity streaks and near-wall vortical structures by Richter and Sullivan (2014). These two characteristics of suspension flows could be seen to, respectively, hasten or delay reattachment, the former by stabilizing the shear layer (Fig. 1b), thereby reducing wallward momentum mixing, and the latter by rapidly diffusing the shear layer’s vortical structures (Fig. 1c), thereby promoting wallward momentum transfer. The fast pressure-recovery rates and short reattachment lengths broadly observed here across all STS cases would suggest that the shear layer is reattaching quickly. It is unlikely that the shear layer is becoming increasingly unstable within the STS cases, as previous studies have shown that the suspended phase acts to attenuate coherent vortical structures that would otherwise act to destabilize the flow. Instead, it is more likely that the shear layer reattaches quickly due to the suspended phase contributing to the rapid diffusion of the shear layer and the underlying vortical structures, causing the flow to reattach more quickly due to increased momentum transfer toward the wall. The rapid diffusion of vortical structures has been observed in previous studies on separated flow within dense suspensions (Barnes et al. 2024; Zhang and Rival 2020).

This rapid diffusion of the shear layer suggested by the pressure and ultrasound measurements would suggest that turbulence within STSs at high volume fractions is prone to the “leveling-out” behavior seen in Newtonian dense suspensions. Furthermore, the underlying coherent structures should appear larger and more dilute, as has been observed in previous studies. However, although the reported results point toward the rapid diffusion of the shear layer and the expansion of the underlying vortical structures, these statements cannot be said conclusively without access to improved flow-field data. The ultrasound failed to penetrate deeper into the pipe due to reflections caused by sudden changes in density between the carrier phase, the suspended phase and the trapped air bubbles. Alternative experimental methods such as X-ray particle tracking velocimetry (XPTV; see Aliseda and Heindel (2021)) should be considered to further investigate onerous suspensions such as the ones tested here.

## Conclusions

Motivated by the need to better characterize turbulent coherent structures in shear-thinning suspensions, the current study experimentally investigated the reattachment behavior of shear layers forming downstream of a sudden expansion with an expansion ratio of 0.5. Four fluids were tested: water; 1750 ppm xanthan-gum-in-water solution (XG${\Phi }0\%$); and two shear-thinning suspensions comprised of 1750 ppm xanthan-gum-in-water solution loaded with non-reactive mineral microspheres to volume fractions of 15% and 30% (XG${\Phi }15\%$ and XG${\Phi }30\%$, respectively). The reattachment behavior of the four fluids were explored through pressure measurements and ultrasound-image velocimetry (UIV) taken from 0*h* to 25.8*h* and 1*h* to 9*h* downstream of the expansion’s outlet, respectively. Here, *h* represents the expansion’s step height and represents the difference between the pipe and throat radii.

The pressure-recovery profiles and the reattachment lengths of the tested single-phase fluids were consistent with single-phase shear-layer behavior observed in previous studies. Specifically, the single-phase pressure-recovery rates were shown to rise as ${\mathrm{Re}}_{\mathrm{eff}}$ increased from ${O}(10^2)$ to ${O}(10^3)$ and stabilize when ${\mathrm{Re}}_{\mathrm{eff}}$ reached ${O}(10^3)$ to ${O}(10^4)$. Correspondingly, large reattachment lengths were observed for the single-phase fluids when ${\mathrm{Re}}_{\mathrm{eff}}$ was ${O}(10^2)$, which then reduced to roughly 8*h* when ${\mathrm{Re}}_{\mathrm{eff}}$ was increased to ${O}(10^3)$. The behavior is consistent with previous studies on single-phase reattachment, which have generally observed how the shear layer: convects further downstream when ${\mathrm{Re}}_{\mathrm{eff}}$ increases toward ${O}(10^2)$, resulting in a delayed pressure recovery and an elongated recirculation zone; increasingly destabilizes as ${\mathrm{Re}}_{\mathrm{eff}}$ increases from ${O}(10^2)$ to ${O}(10^3)$, which quickens pressure recovery and shortens the recirculation zone; and finally exhibits a reattachment length and pressure recovery that is independent of ${\mathrm{Re}}_{\mathrm{eff}}$ within the self-similar turbulent regime.

In contrast, with regard to the tested shear-thinning suspensions, their pressure-recovery profiles and reattachment lengths were inconsistent with single-phase shear-layer behavior. The pressure-recovery profiles of XG${\Phi }15\%$ and XG${\Phi }30\%$ showed no Reynolds-number dependence, and, respectively, matched and overtook the pressure recovery of single-phase fluids within the self-similar turbulent regime. The result agreed with UIV pipe-wall velocity measurements for XG${\Phi }15\%$, which also showed no Reynolds-number dependence and, based on sign change, indicated a reattachment length roughly equal to that of the single-phase fluids within the turbulent regime.

The rapid pressure recovery and short reattachment lengths of the shear-thinning suspensions suggest that the suspended phase within the shear-thinning suspensions does not stabilize the shear layer through coherent-structure attenuation, which would promote an elongated reattachment length and delay pressure recovery by reducing wallward momentum exchange. Instead, it is believed that the suspended phase acts to “level-out” turbulence, which rapidly diffuses the shear layer causing it to reattach sooner due to increased momentum transfer toward the wall. The rapid diffusion of vortical structures has been observed in previous studies on separated flows in dense suspensions. However, the authors refrain from stating this finding as conclusive, given the current study’s inability to capture full flow fields within the shear-thinning suspensions. Alternative measurements, such as XPTV, should be attempted in the future when investigating flows in suspensions with significant density discontinuities such as the ones considered here, a pursuit which is left as future work.

## Data Availability

Data are available upon request.
